# Prevalence of primary Sjögren’s syndrome in patients undergoing evaluation for pulmonary arterial hypertension

**DOI:** 10.1371/journal.pone.0197297

**Published:** 2018-05-15

**Authors:** Tatsuyuki Sato, Masaru Hatano, Yukiko Iwasaki, Hisataka Maki, Akihito Saito, Shun Minatsuki, Toshiro Inaba, Eisuke Amiya, Keishi Fujio, Masafumi Watanabe, Kazuhiko Yamamoto, Issei Komuro

**Affiliations:** 1 Department of Cardiovascular Medicine, Graduate School of Medicine, The University of Tokyo, Tokyo, Japan; 2 Department of Therapeutic Strategy for Heart Failure, Graduate School of Medicine, The University of Tokyo, Tokyo, Japan; 3 Department of Allergy and Rheumatology, Graduate School of Medicine, The University of Tokyo, Tokyo, Japan; 4 Laboratory for Autoimmune Diseases, RIKEN Center for Integrative Medical Sciences, Yokohama, Japan; Nagoya University, JAPAN

## Abstract

**Background:**

The prevalence of pulmonary arterial hypertension (PAH) in primary Sjögren’s syndrome (SS) had been reported to be rare. However, recent studies using echocardiography as a screening method showed conflicting results, and the true prevalence is still unclear. Since diagnosing primary SS is difficult because of its heterogeneous nature, a number of patients with primary-SS-associated PAH may be misdiagnosed with idiopathic PAH, losing their chance to undergo immunosuppressive therapy. Therefore, we sought to elucidate the prevalence of primary SS among patients who initially present with PAH.

**Methods:**

From our prospective institutional PAH database, 40 consecutive patients without any obvious cause of PAH at the time of PAH diagnosis were identified. We retrospectively evaluated the prevalence of primary SS diagnosed during or after the initial assessment of PAH.

**Results:**

During the initial assessment, one patient was diagnosed with primary-SS-associated PAH. Among the 25 patients who were initially diagnosed with idiopathic PAH, five were diagnosed with primary SS during their course of the disease. Of the five patients, three had key signs suggesting primary SS and were probably underdiagnosed at the time of initial evaluation. The remaining two patients, who were finally diagnosed with primary SS, did not have any specific signs suggesting primary SS at the time of initial evaluation but showed positive conversion of their autoantibodies during the course of PAH.

**Conclusion:**

The prevalence of primary-SS-associated PAH may be relatively high among patients who undergo initial evaluation for PAH. Furthermore, primary-SS-associated PAH may be underdiagnosed with routine evaluation for the primary cause of PAH. Clinicians should pay specific attention and carefully evaluate the possibility of primary SS in patients with PAH.

## Introduction

Pulmonary arterial hypertension (PAH) is one of the complications of connective tissue diseases (CTDs), such as systemic sclerosis, systemic lupus erythematosus, and mixed connective tissue disease. The prevalence of PAH and efficacy of immunosuppressive therapies vary depending on the underlying CTDs, but a proportion of patients with CTD-associated PAH is known to respond to immunosuppressive therapies [1]. Because some patients with CTD-associated PAH initially present with pulmonary hypertension, mimicking idiopathic PAH [2], suspicion and accurate diagnosis of CTDs in patients who were diagnosed with pulmonary hypertension is important for clinicians/clinical physicians.

Primary Sjögren’s syndrome (SS) is a systemic autoimmune disease that affects the exocrine glands, such as the salivary and lacrimal glands, and typically presents with sicca symptoms. PAH had been reported as a rare complication of primary SS [3], but recent studies using echocardiography as a screening method suggest that a substantial proportion of patients with primary SS may develop PAH [4,5], and the true prevalence of PAH among patients with primary SS is still undetermined. Because specific attention is often required for accurate primary SS diagnosis due to the existence of patients with primary SS without sicca symptoms [6], a proportion of patients with primary-SS-associated PAH may be misdiagnosed with idiopathic PAH, losing their chance to undergo immunosuppressive therapies. Therefore, we sought to elucidate the prevalence of primary SS among patients who initially present with PAH.

## Methods

### Study design and patient population

From our prospective institutional pulmonary hypertension database, patients who were admitted to our hospital between January 2005 and August 2016 were retrospectively identified. Patients who were finally diagnosed as group 1 or 1’ pulmonary hypertension based on the classification of the 5th World Symposium were included in the present study [7]. Those who were diagnosed with PAH during the follow-up of a primary disease and those who were younger than 18 years at the time of PAH diagnosis were excluded. Those without any obvious cause of PAH before PAH diagnosis consisted the present study population. We retrospectively evaluated the prevalence of primary SS diagnosed during or after the initial assessment of PAH. The study was conducted in accordance with the Declaration of Helsinki, and was approved by the Medical Ethics Committee of the University of Tokyo (No. 2650-(4)). Because this was a retrospective descriptive study with no intervention, and figures do not show identifiable patients, opt-out method was used to obtain consent, with the approval of the Medical Ethics Committee of the University of Tokyo.

### Diagnosis of primary SS

All the medical records of the included patients were reviewed by an experienced physician for evaluation of the possibility of primary SS. Primary SS was diagnosed based on the 2016 American College of Rheumatology (ACR)/European League Against Rheumatism (EULAR) classification criteria [8]. Those who fulfilled the criteria were defined as definite primary SS, and those who partially met the criteria (except for those with only positive anti-SSA/Ro antibody) were defined as possible primary SS. Positive skin biopsy tests were deemed equal to positive lip biopsy tests [9]. All patients who were diagnosed as definite or possible primary SS were consulted by a rheumatologist to confirm the diagnosis and that SS is not secondary to other CTDs.

### Data collection and assessments

Baseline patient demographics, laboratory data, and hemodynamic parameters during PAH and primary SS diagnoses were retrospectively collected during the review of medical records. The parameters of patients who were diagnosed with definite or possible primary SS were compared with those of patients with idiopathic PAH without primary SS. In patients with primary-SS-associated PAH who had intensive immunosuppressive therapy (prednisolone 1 mg/kg/day for 4 weeks followed by intravenous cyclophosphamide 500 mg/m^2^ every 4 weeks for 6 courses), the hemodynamic, physiological, and laboratory parameters before, after the initial 4-week prednisolone therapy, and after completion of the final intravenous cyclophosphamide courses were also collected and evaluated.

### Statistical analysis

Categorical variables were presented as the number of patients (corresponding percentages) and were compared using the Fisher’s exact test. Continuous variables were expressed as the mean±standard deviation or median (interquartile range), depending on its distribution, as assessed by visual inspection and Shapiro–Wilk test, and were compared using Mann–Whitney U test. P<0.05 was considered statistically significant, and all P-values were two sided. All statistical analyses were performed using JMP pro 12.2 (SAS Institute Inc., Cary, NC, USA).

## Results

During the study period, 132 patients with PAH were admitted to our hospital. Of them, 56, 19, 10, and 1 patients were diagnosed with PAH during the follow-up of CTDs, congenital heart disease, hepatic disease, or hematologic disease, respectively. Six patients were excluded because they were diagnosed with PAH at age <18 years. This resulted in 40 patients (30.3%) consisting the present study population. Table 1 shows the baseline patient demographics of the study population. All patients were Japanese, except one Burmese patient.

**Table 1:** Baseline patient characteristics at the time of PAH diagnosis.

Total number of patients	40
Female sex	28 (70)
Age, years	37±13
Body mass index, kg/m^2^	22±4
Smoking history	18 (45)
Coexisting conditions	
Hypertension	4 (10)
Diabetes mellitus	2 (5)
Modified NYHA/WHO functional class	
I	2 (5)
II	12 (30)
III	20 (50)
IV	6 (15)
Time from onset to diagnosis, months	4 (1–13)
Mean PAP, mm Hg	54±15
Cardiac index, L/min/m^2^	2.4±0.8

Numbers are reported as n (%), mean±standard deviation, or median (interquartile range). PAH, pulmonary arterial hypertension; PAP, pulmonary artery pressure.

**Fig 1:**
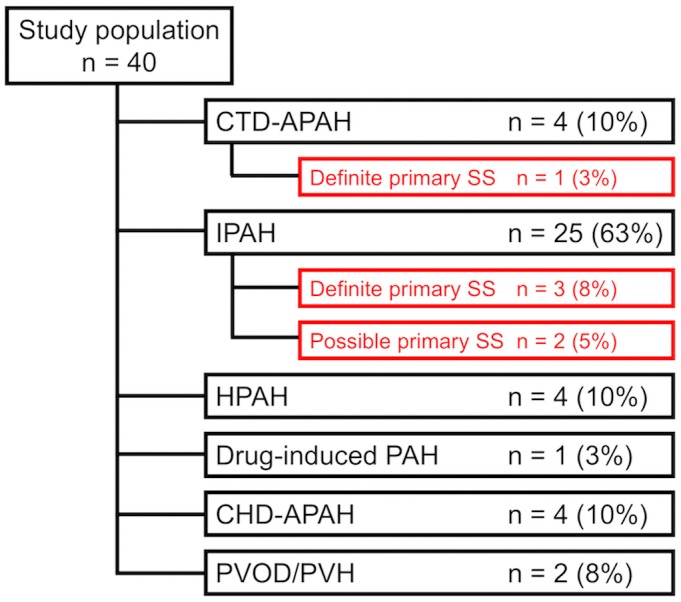
Initial diagnosis and prevalence of primary Sjögren’s syndrome.

Fig 1 shows the primary diagnosis at the time of PAH presentation and prevalence of primary SS among the study population. One patient was initially diagnosed with definite primary SS and primary-SS-associated PAH at the initial evaluation. Among the 25 patients initially diagnosed with idiopathic PAH, three were diagnosed with definite primary SS years after PAH diagnosis, and two met the criteria for possible primary SS during their course of PAH.

Among the study population, one patient was initially diagnosed with definite primary SS and associated PAH. Twenty-five patients were initially diagnosed with IPAH, and among them, three and two patients were diagnosed with definite and possible primary SS during their course of the disease, respectively. CHD-APAH, congenital heart disease-associated pulmonary arterial hypertension; CTD-APAH, connective tissue disease-associated pulmonary arterial hypertension; HPAH, hereditary pulmonary arterial hypertension; IPAH, idiopathic pulmonary arterial hypertension; PAH, pulmonary arterial hypertension; PVH, pulmonary venous hypertension; PVOD, pulmonary veno-occlusive disease.

S1 Table shows the matched and the unmatched items of the diagnostic criteria in each patient. Because unstimulated salvia test was not performed in any of the patients, the results of gum test were additionally collected. Table 2 summarizes the clinical courses of the six patients with primary SS. The patients were all female. Patient 1 was initially diagnosed with definite primary SS and primary-SS-associated PAH. The patient had increased anti-SSA/Ro antibody level and mildly positive anti-nuclear antibody (ANA) of 1:40 during PAH diagnosis. She had no sicca symptoms, but her gum test was positive. Salivary gland scintigraphy suggested decreased salivary gland function, and lip biopsy revealed typical lymphocytic sialadenitis. She was diagnosed with definite primary SS along with primary-SS-associated PAH and underwent immunosuppressive therapy. Patient 2 was diagnosed with primary biliary cirrhosis 2 years before the PAH diagnosis. She was initially diagnosed with idiopathic PAH, but the evaluation of sicca symptoms, including medical interview, or the evaluation of anti-SSA/Ro antibody was not performed. Her pulmonary artery pressure gradually increased and she was transferred to our hospital two years after the initial diagnosis. She was not taking any PAH specific treatment at the time of the transferal. Focused medical interview revealed mild sicca symptoms for more than two years. She also had positive anti-SSA/Ro antibody and positive lip biopsy results, resulting in the diagnosis of definite primary SS with primary-SS-associated PAH, and she underwent immunosuppressive therapy. Patient 3 already had a positive anti-SSA/Ro antibody during PAH diagnosis but because sicca symptoms were absent, she was initially diagnosed with idiopathic PAH and had started epoprostenol, without any further examinations focusing on primary SS. A year later, she had a rash and was diagnosed with definite primary SS via skin biopsy. However, the association between primary SS and PAH was deemed scarce, and she was not diagnosed with primary-SS-associated PAH and did not undergo immunosuppressive therapy. Patient 4 had no sicca symptoms, and her anti-SSA/Ro antibody test was negative during idiopathic PAH diagnosis. However, 5 years later, her test for anti-SSA/Ro antibody showed positive. She had negative lip biopsy results, but Schirmer’s test result was positive, and she was diagnosed with definite primary SS. Because her pulmonary artery pressure was under control with conventional medical therapy including treprostinil, bosentan, sildenafil, tadalafil, and beraprost, she did not undergo immunosuppressive therapy. Patient 5 was initially diagnosed with idiopathic PAH because she had no sicca symptoms and no anti-SSA/Ro antibody during the initial evaluation, although she was tested positive for ANA. Her pulmonary artery pressure was under control with tadalafil, macitentan, and beraprost. She eventually developed sicca symptoms, and Schirmer’s test, gum test, and salivary gland scintigraphy performed on her 3 years after PAH diagnosis showed positive results. Patient 6 had increased ANA levels and hypergammaglobulinemia after 9 years of idiopathic PAH diagnosis and was suspected of primary SS. She had been taking epoprostenol, ambrisentan, and tadalafil for the treatment of PAH. She underwent salivary gland scintigraphy and Schirmer’s test, both of which showed positive results. Although both patients 5 and 6 had been diagnosed with possible primary SS, they did not undergo lip biopsy to confirm the diagnosis of primary SS nor further treatment with intensive immunosuppression because their pulmonary artery pressures were controlled with conventional therapies at the time of primary SS diagnosis. In total, ANA sensitivity for primary SS diagnosis was 67%, but half of the ANA-positive patients were only weakly positive. All but one of the ANA measurements shown in Table 2 were performed using indirect immunofluorescence assay (IFA) with human epithelial type 2 (HEp-2) cells. The method for ANA measurement during PAH diagnosis in one patient was unknown because she underwent initial evaluation for PAH at another institution years before the present study, and the data was unavailable.

**Table 2:** Summary of patients with primary SS.

Patient No.	Diagnosis of primary SS	At the time of PAH diagnosis						Diagnosis of SS at the time of PAH diagnosis	Months from PAH diagnosis to SS diagnosis	At the time of primary SS diagnosis						
		Age, years	Sicca symptoms	ANA ^a^	Anti SSA/Ro antibody	Anti SSB/La antibody	Comorbid Conditions			Sicca symptoms	ANA ^a^	Anti SSA/Ro antibody	Anti SSB/La antibody	Schirmer’s test	Gum test^c^ [10]	Biopsy
1	Definite	32	None	Speckled 1:40	Positive	Negative	None	Yes	0				Negative	Negative	Positive	Positive
2	Definite	45	Not evaluated	Not evaluated	Not evaluated	Not evaluated	PBC	No	27	Positive	Negative	Positive	Negative	Negative	Positive	Positive
3	Definite	19	Negative	NA	Positive	Positive	None	No	18	Positive	NA	Positive	Positive	NA	NA	Positive^d^
4	Definite	49	Negative	Negative	Negative	Negative	None	No	64	Negative	Negative	Positive	Negative	Positive	Negative	Negative
5	Possible	55	Negative	Positive ^b^ 1:160	Negative	Negative	None	No	40	Positive	Granular 1:320	Negative	Negative	Positive	Positive	Not performed
6	Possible	37	Not evaluated	Negative ^b^	Not evaluated	Not evaluated	None	No	117	Negative	Positive ^b^ 1:1280	Negative	Negative	Positive	Negative	Not performed

All the patients were female. NA, not available, indicates that whether the actual test had been performed or not is unknown. ANA, anti-nuclear antibody; PAH, pulmonary arterial hypertension; SS, Sjögren’s syndrome; primary-SS-APAH, primary-Sjögren’s-syndrome-associated pulmonary arterial hypertension

^a^Method used for ANA detection was immunofluorescence test using HEp-2 cells, unless otherwise specified.

^b^Neither the method used for ANA detection nor the fluorescence pattern of ANA was unknown.

^c^Gum test result of <10 mL/10 min. was considered positive.

^d^Skin biopsy.

Table 3 shows the summary of the severity of the disease in patients with primary SS. Data of patient 3 were unavailable because primary SS diagnosis was made in another institution more than 10 years ago, and medical records were unavailable. All other patients whose data were available had low ESSDAI score. Individual data used for the calculation of ESSDAI score are listed in S2 Table. Mild hypergammaglobulinemia was present in patients 2 and 6. Neither Raynaud symptoms nor interstitial lung disease was present in any patient.

**Table 3:** Disease severity of patients with primary SS.

Patient No.	1	2	4	5	6
ESSDAI	3	1	0	0	3
IgG, ng/dL	1142	1875	919	1020	1995
sIL-2R, U/mL	377	540	NA	NA	449
Ferritin, ng/mL	53	NA	NA	6	12
Raynaud symptom	–	–	–	–	–
Interstitial lung disease	–	–	–	–	–

Reference ranges: IgG, 861–1747 ng/dL; sIL-2R, 127–582 U/mL; ferritin, 5–152 ng/mL. ESSDAI, European League Against Rheumatism Sjögren’s Syndrome Disease Activity Index; NA, not available; sIL-2R, soluble interleukin-2 receptor.

Table 4 shows the comparison of the characteristics of the patients who were diagnosed with primary SS during their course of the disease and that of the patients who were diagnosed with idiopathic PAH without primary SS. The baseline characteristics did not differ significantly between the groups. Patients with primary-SS-associated PAH were all female.

**Table 4:** Comparison of characteristics between patients with primary SS and idiopathic PAH patients without primary SS.

	primary SS	Non-primary-SS IPAH	P
	(n = 6)	(n = 20)	
Female sex	6 (100)	12 (60)	0.13
Age at PAH diagnosis, years	39.5±13.0	32.0±10.7	0.17
WHO functional class			0.41
I	0 (0)	1 (5)	
II	1 (17)	7 (35)	
III	5 (83)	8 (40)	
IV	0 (0)	4 (20)	
Time from onset to diagnosis, months	2.5 (0.0–6.0)	4.5 (1.0–22.5)	0.39
Mean PAP, mm Hg	55.0±9.2	55.9±14.4 ^a^	1.00
Cardiac index, L/min/m^2^	2.3±0.6	2.3±0.8^b^	0.48

Numbers are reported as n (%), mean±standard deviation, or median (interquartile range). IPAH, idiopathic pulmonary arterial hypertension; PAH, pulmonary arterial hypertension; PAP, pulmonary artery pressure; SS, Sjögren’s syndrome.

^a^One missing datum.

^b^Two missing data.

Patients 1 and 2 underwent immunosuppressive therapy. Table 5 shows the changes in hemodynamic, physiological, and laboratory parameters of the two patients. Both patients showed improvement in not only hemodynamic parameters, but also in physiological and laboratory parameters after the initial steroid therapy, although no pulmonary vasodilators were prescribed during this period. After the initial steroid therapy, both patients continued treatment with intravenous cyclophosphamide therapy, with favorable outcomes after the completion of the treatment course. After treatment, patients 1 and 2 had been followed up for 2.3 and 3.1 years, respectively, without hospitalization due to heart failure, death, or the need for additional intravenous or subcutaneous pulmonary vasodilators. In contrast, 16 of the 20 patients who were diagnosed with idiopathic PAH without primary SS died or required intravenous pulmonary vasodilators during their treatment course (median follow-up period: 2.0 years (interquartile range, 1.1–8.2 years)).

**Table 5:** Results of immunosuppressive therapy in two patients.

	Patient 1			Patient 2		
	Pre	Post PSL	Post IVCY	Pre	Post PSL	Post IVCY
Mean PAP, mm Hg	57	42	19	33	23	28
PVR, dynes	1177	675	189	446	354	320
Mean PAWP, mm Hg	8	10	6	11	6	12
Cardiac index, L/min/m^2^	1.9	2.2	3.2	2.6	2.6	2.7
SvO2, %	70	74	79	76	78	73
6MWT, m	420	420	NA	300	560	560
BNP, pg/mL	359.9	59.0	21.6	28.4	8.4	10.3
IgG, ng/dL	1142	851	866	1875	1061	737
Simultaneous use of pulmonary vasodilators	No	No	Yes ^a^	No	No	No

Immunosuppressive therapy protocol: prednisolone 1 mg/kg/day for 4 weeks followed by intravenous cyclophosphamide 500 mg/m^2^ every 4 weeks for 6 courses.

BNP, brain natriuretic peptide; IVCY, intravenous cyclophosphamide; PAP, pulmonary artery pressure; PAWP, pulmonary arterial wedge pressure; PSL, prednisolone; PVR, pulmonary vascular resistance; 6MWT, 6-min walk test.

^a^Tadalafil 40 mg/day and bosentan 250 mg/day.

## Discussion

The present study was the first to evaluate the prevalence of primary SS among patients with PAH who have no known primary diseases. The most significant finding of the present study was that five patients met or partially met the criteria for primary SS during their course of the disease among the 25 patients who were initially diagnosed with idiopathic PAH. Although only one was a reported case of primary-SS-associated PAH wherein PAH diagnosis preceded primary SS diagnosis [11], the results of our study suggest that a proportional number of patients with PAH is diagnosed with primary SS years after the initial PAH diagnosis.

Among the five patients with primary SS, three already had sicca symptoms, positive ANA, positive anti-SSA/Ro antibody, or history of primary biliary cirrhosis during the initial evaluation for PAH. They did not undergo sufficient evaluation at that time, suggesting that primary SS had been underdiagnosed during the initial evaluation. There could be three major explanations for their underdiagnoses of primary SS.

First, some patients with primary SS have no or only mild sicca symptoms, and primary SS diagnosis is difficult to suspect only from general history taking or physical examinations. In our study, sicca symptoms were absent during primary SS diagnosis in half of the cases, but they all had positive Schirmer’s or Gum test results. A similar study reported by Kurumagawa *et al*. showed that, among 313 patients with diffuse lung disease who had neither primary xerosis nor Sjögren’s syndrome, 54 (17.3%) had decreased saliva production, which was detected by Saxon test, and 29 (9.3%) were diagnosed with definite or probable Sjögren’s syndrome [12]. These results support the hypothesis that a non-negligible number of patients with PAH have subclinical Sjögren’s syndrome. Clinicians should conduct specific history taking focusing on sicca symptoms and may consider routine use of simple objective tests, such as Schirmer’s, Saxon, or Gum test for screening of xerostomia and xerophthalmia upon the initial evaluation of PAH.

Second, some clinicians may be excluding primary SS because of negative ANA results, which only have moderate sensitivity for primary SS diagnosis. In the present study, the sensitivity of ANA for primary SS diagnosis was 67% and 33%, with a cutoff dilution of 1:40 and 1:80, respectively. This was even lower than the sensitivity of ANA for primary SS diagnosis in general: 81.2%–84.2% and 76.3% with a cutoff dilution of 1:40 and 1:80, respectively [13, 14]. ANA has been reported to have low sensitivity in primary SS diagnosis in general because the gold standard method for ANA measurement, IFA, lacks sensitivity for the detection of anti-SSA/Ro antibody [15]. Because the present study consisted of atypical patients whose initial presentation was PAH, the sensitivity may have been even lower. In the present study, 2 of 4 patients with primary SS with ANA titer <1:80 had positive anti-SSA/Ro antibody. ANA was excluded from the most recent international criteria for primary SS approved by ACR and EULAR because of its poor utility over anti-SSA/Ro antibody [8,16]. Although the sensitivity of anti-SSA/Ro antibody for primary SS diagnosis is approximately 80% and is not adequate for exclusion of primary SS [13], our data suggest that the evaluation of ANA only is insufficient, and that anti-SSA/Ro antibody testing should be added for primary SS screening in patients with PAH.

In terms of ANA, remembering that the above discussion only applies to the ANA measurement with IFA using the most common substrate, HEp-2 cells, is important. Because HEp-2 cells lack SSA/Ro antigen, ANA measurement with IFA using HEp-2 cells has low sensitivity in primary SS diagnosis [17]. HEp-2000 cells, which are HEp-2 cells transfected with Ro60 cDNA, have been developed to improve sensitivity for detecting anti-SSA/Ro antibody and are now being used in daily practice [17]. Although it is not fully evaluated, ANA measurement using HEp-2000 may have better sensitivity in primary SS diagnosis. ANA measurement by enzyme-linked immunosorbent assay (ELISA) may also be another method with increased sensitivity for primary SS. ELISA is now being widely used for the initial evaluation of ANA because it is less expensive than IFA, and can be easily automated with no need for experienced technologists to read and interpret the assay. Numerous ELISA kits for ANA detection are commercially available, and most of them have SSA/Ro as an antigen, which means improved sensitivity for primary SS diagnosis [18]. However, the sensitivity of ANA measured with ELISA for other CTD diagnoses, such as systemic lupus erythematosus, has been reported to be inferior to that of ANA measured with IFA [19], and ACR recommends IFA as a gold standard for ANA detection [20]. Understanding which method is being used in their institution to correctly interpret the results of ANA testing is important for clinicians.

Third, the activity of primary SS can be low in some primary-SS-associated PAH cases, avoiding the clinicians to perceive the true association between the two diseases and to proceed to further evaluation focusing on primary SS. In the present study, all patients with primary SS whose data at the time of primary SS diagnosis were available had ESSDAI score <5. ESSDAI is a score developed by EULAR to assess systemic complications of primary SS [21], and score <5 indicates low disease activity. In addition, other parameters suggesting disease activities were also absent in majority of our primary SS cases. This result was conflicting with the previous study by Launay *et al*. which showed that almost 80% of the 28 reported patients with primary-SS-associated PAH had hypergammaglobulinemia [3]. This may be because in the previous study, 26 of the 28 patients were diagnosed with primary SS preceding or concomitant with PAH diagnosis, whereas five of the six patients in the present study were diagnosed with PAH preceding primary SS diagnosis. A report by Reina *et al*. has shown that among 25 patients with primary SS with interstitial lung disease, 15 were diagnosed with interstitial lung disease preceding primary SS diagnosis, and majority of the patients had low ESSDAI score [22]. Although the assessment of disease severity has been reported to be important to predict lethal complications in patients who are already diagnosed with primary SS [23], it may be of less use in atypical patients with primary SS who initially present with pulmonary hypertension.

Of course, the true pathophysiologic association between the low-active primary SS and PAH is still to be elucidated. However, in the present study, immunosuppressive therapy was effective in two patients with PAH with primary SS, both having a low ESSDAI score with no or only mild hypergammaglobulinemia. This result suggests the presence of autoimmune aspects in the pathophysiology of PAH in these patients with low-active primary SS. We should not exclude primary-SS-associated PAH in a PAH patient with primary SS because of low disease activity and should not hesitate to initiate immunosuppressive therapy.

The mechanisms of PAH itself is still unclear and that of CTD-associated PAH is much less understood. To the best of our knowledge, there are no primary SS specific model mouse exhibiting PAH. MRL/lpr mouse, a model mouse of SS and systemic lupus erythematosus, has been reported to develop pulmonary hypertension and the phosphorylation disorder of eNOS, elevation of prepro-endothelin-1, and the upregulation of survivin were considered as possible molecular mechanisms [24]. Data are still scarce and further studies are required to elucidate the mechanisms and possible presentations of primary-SS-associated PAH.

Other than these three patients with primary SS probably underdiagnosed during the initial evaluation, the remaining two cases had positive conversion of their autoantibodies after PAH diagnosis and had been diagnosed as definite or possible primary SS. The clinical course of these two patients may be showing not just a simple complication of primary SS in patients with primary-SS-unassociated idiopathic PAH, but a seroconversion of seronegative primary-SS-associated PAH. Although no data of autoantibody seroconversion were found in patients with primary SS, a study of systemic lupus erythematosus had shown that autoantibodies do fluctuate during the course of the disease [25]. Because the two patients in our study did not undergo immunosuppressive therapy, we cannot state from our data whether the PAH of these patients are associated with primary SS. However, this result may suggest the importance of periodic reappraisal of autoantibodies in patients who were diagnosed with idiopathic PAH to detect underlying CTDs.

One of the two patients had been given intravenous epoprostenol at the time of SS diagnosis. Epoprostenol treatment has been reported to be associated with autoimmune thyrotoxicosis, and may also cause other autoimmune diseases such as SS [26, 27]. However, to the best of our knowledge, there are no reported case of primary SS being diagnosed after epoprostenol treatment. Whether it is not happening or that we are just underdiagnosing SS is still not clear. Further studies are warranted to clarify the association between epoprostenol treatment and SS.

In the present study, 1 of the 40 patients (2.5%) was initially diagnosed as SS-associated PAH. This result is consistent with a similar study reported by Cavagna *et al*., showing that only 1 out of 49 suspected idiopathic PAH patients (2.0%), have met the criteria of SS syndrome at the time of the initial evaluation [28]. We have additionally found that 5 out of the 25 patients who were initially diagnosed with idiopathic PAH was later diagnosed as primary SS. This result may emphasize the importance of repeat evaluation of undiagnosed CTDs in patients who are diagnosed and being followed up as idiopathic PAH. The efficacy of immunosuppressive therapies in lately-CTD-diagnosed idiopathic PAH patients has not been evaluated and further studies are warranted.

The present study had several limitations. First, the patients included in this study were all Japanese, except for one Burmese patient. Several cohort studies conducted in Japan and China have reported that the prevalence of primary-SS-associated PAH is comparable to that of SSc-associated PAH, and that primary SS may be one of the major primary causes for CTD-associated PAH [29–31]. Meanwhile, cohorts from Europe and the United States have shown that only a negligible number of patients have primary-SS-associated PAH [32, 33]. Moreover, the prevalence of Japanese patients among the reported primary-SS-associated PAH cases is high [34]. The prevalence of primary-SS-associated PAH may differ between races, and the results of the present study may not be extrapolated to other races. Second, the present study was a retrospective single-center study with a small sample size. Our institution is a referral center for pulmonary hypertension, and a referral bias may be present. The initial diagnosis of PAH had been made in other institutions more than 10 years prior in some cases, and the available information was limited in those cases. The results should be considered preliminary, and further studies are warranted.

A proportional number of patients with primary-SS-associated PAH may be misdiagnosed with idiopathic PAH. Clinicians should carefully evaluate the possibility of primary SS in their initial evaluation of PAH.

## Supporting information

S1 Table Fulfilment of 2016 EULAR/ACR criteria. (DOCX)

S2 Table Individual data used for the calculation of ESSDAI score. (DOCX)
