# Supplementary material for: Prevalence of primary Sjögren’s syndrome in patients undergoing evaluation for pulmonary arterial hypertension
Source: PLoS One. 2018 May 15;13(5):e0197297. doi: 10.1371/journal.pone.0197297 (PMC5953489; doi:10.1371/journal.pone.0197297)
Supplement: S1 Table — (DOCX) [file pone.0197297.s001.docx]

Supplementary table 1. Fulfilment of 2016 EULAR/ACR criteria

| Patient No. | 1 | 2 | 3 | 4 | 5 | 6 |
| --- | --- | --- | --- | --- | --- | --- |
| Labial salivary gland with focal lymphocytic sialadenitis and focus score of ≥1 foci/4 m^2^ | positive | positive | positive^a^ | negative | NA | NA |
| anti-SSA/Ro positive | positive | positive | positive | positive | negative | negative |
| Ocular Staining Score ≥5 (or van Bijsterveld score ≥4) in at least 1 eye | negative | negative | NA | negative | positive | negative |
| Schirmer's test ≤5 mm/5 minutes in at least 1 eye | negative | negative | NA | positive | positive | positive |
| Unstimulated whole saliva flow rate ≤0.1 mL/minute | NA | NA | NA | NA | NA | NA |
| Total Score | 6 | 6 | 6 | 4 | 2 | 1 |

NA, not available, indicates that whether the actual test had been performed or not is unknown.

^a^Skin biopsy.
