# Supplementary material for: Prevalence of primary Sjögren’s syndrome in patients undergoing evaluation for pulmonary arterial hypertension
Source: PLoS One. 2018 May 15;13(5):e0197297. doi: 10.1371/journal.pone.0197297 (PMC5953489; doi:10.1371/journal.pone.0197297)
Supplement: S2 Table — (DOCX) [file pone.0197297.s002.docx]

Supplementary table 2. Individual data used for the calculation of ESSDAI score

| Patient No. | 1 | | 2 | 4 | | 5 | | 6 | |
| --- | --- | --- | --- | --- | --- | --- | --- | --- | --- |
| ESSDAI score | 3 | | 1 | 0 | | 0 | | 3 | |
| Urine protein, g/gCre | NA | | 0.18 | NA | | 0.04 | | 0.09 | |
| Urine protein, strip test | negative | | negative | negative | | negative | | negative | |
| Creatinine kinase, U/L | 47 | | 113 | 45 | | 52 | | 62 | |
| Neutrophil count, /mm^3^ | 3600 | | 4700 | 2400 | | 5000 | | 3200 | |
| Lymphocyte count, /mm^3^ | 1700 | | 2400 | 1700 | | 3500 | | 1600 | |
| Hemoglobin, g/dL | 14.0 | | 13.5 | 14.8 | | 13.6 | | 12.1 | |
| Platelet count, /mm^3^ | 137000 | | 188000 | 215000 | | 425000 | | 137000 | |
| C3, mg/dL | 92 | | 108 | 122 | | NA | | 78 | |
| C4, mg/dL | 19 | | 8 | 28 | | NA | | 19 | |
| CH50, mg/dL | 10 | | 52 | 60 | | NA | | 52 | |
| IgG, mg/dL | 1142 | 1875 | | | 919 | | 1020 | | 1995 |

NA, not available. Reference range: Creatinine kinase, 41–153 U/L.
